# 3-Methyl­butyl 2-(5-iodo-3-methyl­sulfinyl-1-benzofuran-2-yl)acetate

**DOI:** 10.1107/S1600536809011210

**Published:** 2009-03-31

**Authors:** Hong Dae Choi, Pil Ja Seo, Byeng Wha Son, Uk Lee

**Affiliations:** a Department of Chemistry, Dongeui University, San 24 Kaya-dong Busanjin-gu, Busan 614-714, Republic of Korea; b Department of Chemistry, Pukyong National University, 599-1 Daeyeon 3-dong, Nam-gu, Busan 608-737, Republic of Korea

## Abstract

In the title mol­ecule, C_16_H_19_IO_4_S, the O atom and the methyl group of the methyl­sulfinyl substituent lie on opposite sides of the plane of the benzofuran fragment. In the crystal, pairs of mol­ecules are linked by I⋯O [3.114 (3) Å] halogen bonding into centrosymmetric dimers. The crystal structure is further stabilized by weak inter­molecular C—H⋯O nonclassical hydrogen bonds.

## Related literature

For the crystal structures of similar alkyl 2-(5-iodo-3-methyl­sulfinyl-1-benzofuran-2-yl)acetate derivatives, see Choi *et al.* (2009*a* ▶,*b* ▶). For a review of halogen bonding, see Politzer *et al.* (2007 ▶).
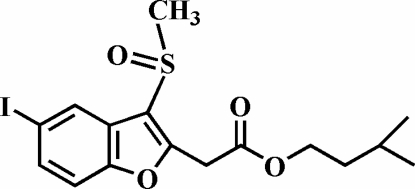


## Experimental

### 

#### Crystal data

C_16_H_19_IO_4_S
*M* *_r_* = 434.27
Monoclinic, 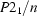

*a* = 10.6726 (9) Å
*b* = 15.423 (1) Å
*c* = 10.7343 (9) Å
β = 102.334 (2)°
*V* = 1726.1 (2) Å^3^
*Z* = 4
Mo *K*α radiation
μ = 1.99 mm^−1^
*T* = 173 K
0.40 × 0.40 × 0.30 mm

#### Data collection

Bruker SMART CCD diffractometer
Absorption correction: multi-scan (*SADABS*; Sheldrick, 1999 ▶) *T*_min_ = 0.503, *T*_max_ = 0.587 (expected range = 0.472–0.550)
9111 measured reflections
3357 independent reflections
3152 reflections with *I* > 2σ(*I*)
*R*_int_ = 0.026

#### Refinement

*R*[*F* ^2^ > 2σ(*F* ^2^)] = 0.034
*wR*(*F* ^2^) = 0.079
*S* = 1.23
3357 reflections
202 parameters
H-atom parameters constrained
Δρ_max_ = 0.56 e Å^−3^
Δρ_min_ = −0.71 e Å^−3^

### 

Data collection: *SMART* (Bruker, 2001 ▶); cell refinement: *SAINT* (Bruker, 2001 ▶); data reduction: *SAINT*; program(s) used to solve structure: *SHELXS97* (Sheldrick, 2008 ▶); program(s) used to refine structure: *SHELXL97* (Sheldrick, 2008 ▶); molecular graphics: *ORTEP-3* (Farrugia, 1997 ▶) and *DIAMOND* (Brandenburg, 1998 ▶); software used to prepare material for publication: *SHELXL97*.

## Supplementary Material

Crystal structure: contains datablocks global, I. DOI: 10.1107/S1600536809011210/cv2534sup1.cif

Structure factors: contains datablocks I. DOI: 10.1107/S1600536809011210/cv2534Isup2.hkl

Additional supplementary materials:  crystallographic information; 3D view; checkCIF report

## Figures and Tables

**Table 1:** Hydrogen-bond geometry (Å, °)

*D*—H⋯*A*	*D*—H	H⋯*A*	*D*⋯*A*	*D*—H⋯*A*
C5—H5⋯O4^i^	0.95	2.41	3.310 (5)	159
C16—H16*C*⋯O3^ii^	0.98	2.44	3.397 (5)	167

Symmetry codes: (i) 
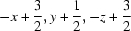
; (ii) 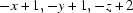.

**Fig. 1.**
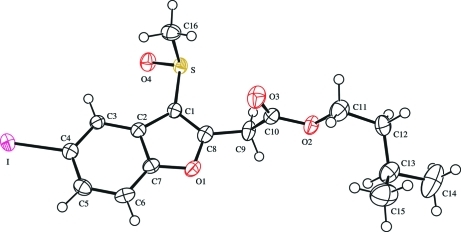
The molecular structure of the title compound with the atom numbering scheme. Displacement ellipsoids are drawn at the 50% probability level. H atoms are presented as a small cycles of arbitrary radius.

**Fig. 2.**
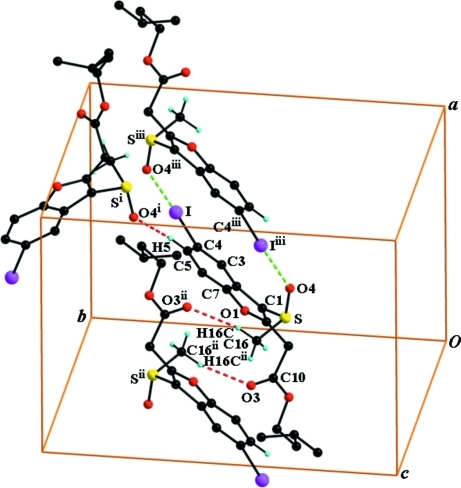
A portion of the crystal packing of (I) showing the C—H···O hydrogen bonds (dashed lines) and I···O halogen bonding (dotted lines) [symmetry codes: (i) -x+3/2, y+1/2, -z+3/2; (ii) -x+1, -y+1, -z+2; (iii) -x+2, -y+1, -z+2]. H atoms not involved in hydrogen bonding are omitted for clarity.

## supplementary crystallographic information

### Comment

As a part of our continuing studies on the synthesis and structure of alkyl 2-(5-iodo-3-methylsulfinyl-1-benzofuran-2-yl)acetate analogues, we have recently described the crystal structure of propyl 2-(5-iodo-3-methylsulfinyl-1-benzofuran-2-yl)acetate (Choi *et al.*,  2009*a*) and butyl 2-(5-iodo-3-methylsulfinyl-1-benzofuran-2-yl)acetate (Choi *et al.*,  2009*b*). Here we report the crystal structure of the title compound, (I) (Fig. 1).

In (I), the benzofuran unit is essentially planar, with the mean deviation of 0.017 (3) Å from the least-squares plane defined by the nine constituent atoms. Two molecules are linked into centrosymmetric dimer by an I···O halogen bonding  (Politzer *et al.*,  2007) between the iodine atom and the oxygen of a neighbouring S═O unit, with an I···O distance of 3.114 (3) Å. The molecular packing (Fig. 2) is stabilized by two intermolecular C—H···O nonclassical hydrogen bonds - between the benzene H atom and the S═O unit, and between the methyl H atom of the methylsulfinyl substituent and the C═O unit, respectively (Fig. 2 and Table 1).

### Experimental

The 77% 3-chloroperoxybenzoic acid (247 mg, 1.1 mmol) was added in small portions to a stirred solution of isoamyl 2-(5-iodo-3-methylsulfanyl-1-benzofuran-2-yl)acetate (434 mg, 1.0 mmol) in dichloromethane (30 ml) at 273 K. After being stirred for 3 h at room temperature, the mixture was washed with saturated sodium bicarbonate solution and the organic layer was separated, dried over magnesium sulfate, filtered and concentrated in vacuum. The residue was purified by column chromatography (hexane-ethyl acetate,1:2 v/v) to afford the title compound as a colorless solid [yield 79%, m.p. 412-413 K; *R*_f_ = 0.53 (hexane-ethyl acetate, 1;2 v/v )]. Single crystals suitable for X-ray diffraction were prepared by evaporation of a solution of the title compound in acetone at room temperature. Spectroscopic analysis: ^1^H NMR (CDCl_3_, 400 MHz) δ 0.91 (d, J = 6.6 Hz, 6H), 1.53 (q, J = 6.6 Hz, 2H), 1.61-1.72 (m, 1H), 3.07 (s, 3H), 4.03 (s, 2H), 4.18 (t, J = 6.96 Hz, 2H), 7.29 (d, J = 8.8 Hz, 1H), 7.66 (dd, J = 8.8 Hz and 1,84 Hz, 1H), 8.28 (d, J = 1.8 Hz, 1H); EI-MS 434 [M^+^].

### Refinement

In the title molecule, C_16_H_19_IO_4_S, the O atom and the methyl group of the methylsulfinyl substituent lie on opposite sides of the plane of the benzofuran fragment. Two molecules are linked by I···O [3.114 (3) Å] halogen bonding into a centrosymmetric dimer. The crystal structure is further stabilized by weak intermolecular C—H···O nonclassical hydrogen bonds.

### Crystal data

**Table:**

C_16_H_19_IO_4_S	*F*(000) = 864
*M**_r_* = 434.27	*D*_x_ = 1.671 Mg m^−^^3^
Monoclinic, *P*2_1_/*n*	Mo *K*α radiation, λ = 0.71073 Å
Hall symbol: -P 2yn	Cell parameters from 7114 reflections
*a* = 10.6726 (9) Å	θ = 2.4–28.3°
*b* = 15.423 (1) Å	µ = 1.99 mm^−^^1^
*c* = 10.7343 (9) Å	*T* = 173 K
β = 102.334 (2)°	Block, colourless
*V* = 1726.1 (2) Å^3^	0.40 × 0.40 × 0.30 mm
*Z* = 4	

### Data collection

**Table:**

Bruker SMART CCD diffractometer	3357 independent reflections
Radiation source: fine-focus sealed tube	3152 reflections with *I* > 2σ(*I*)
graphite	*R*_int_ = 0.026
Detector resolution: 10.0 pixels mm^-1^	θ_max_ = 26.0°, θ_min_ = 2.4°
φ and ω scans	*h* = −13→12
Absorption correction: multi-scan (*SADABS*; Sheldrick, 1999)	*k* = −12→19
*T*_min_ = 0.503, *T*_max_ = 0.587	*l* = −12→13
9111 measured reflections	

### Refinement

**Table:**

Refinement on *F*^2^	Primary atom site location: structure-invariant direct methods
Least-squares matrix: full	Secondary atom site location: difference Fourier map
*R*[*F*^2^ > 2σ(*F*^2^)] = 0.034	Hydrogen site location: difference Fourier map
*wR*(*F*^2^) = 0.079	H-atom parameters constrained
*S* = 1.23	*w* = 1/[σ^2^(*F*_o_^2^) + (0.0195*P*)^2^ + 3.2932*P*] where *P* = (*F*_o_^2^ + 2*F*_c_^2^)/3
3357 reflections	(Δ/σ)_max_ < 0.001
202 parameters	Δρ_max_ = 0.56 e Å^−^^3^
0 restraints	Δρ_min_ = −0.71 e Å^−^^3^

### Special details

**Table:**

Geometry. All esds (except the esd in the dihedral angle between two l.s. planes) are estimated using the full covariance matrix. The cell esds are taken into account individually in the estimation of esds in distances, angles and torsion angles; correlations between esds in cell parameters are only used when they are defined by crystal symmetry. An approximate (isotropic) treatment of cell esds is used for estimating esds involving l.s. planes.
Refinement. Refinement of F^2^ against ALL reflections. The weighted R-factor wR and goodness of fit S are based on F^2^, conventional R-factors R are based on F, with F set to zero for negative F^2^. The threshold expression of F^2^ > 2sigma(F^2^) is used only for calculating R-factors(gt) etc. and is not relevant to the choice of reflections for refinement. R-factors based on F^2^ are statistically about twice as large as those based on F, and R- factors based on ALL data will be even larger.

### Fractional atomic coordinates and isotropic or equivalent isotropic displacement parameters (Å^2^)

**Table:**

	*x*	*y*	*z*	*U*_iso_*/*U*_eq_	
I	0.98665 (2)	0.636473 (16)	0.87775 (2)	0.03496 (10)	
S	0.60639 (9)	0.32117 (6)	0.94130 (8)	0.0275 (2)	
O1	0.4562 (2)	0.47524 (17)	0.6559 (2)	0.0306 (6)	
O2	0.1263 (2)	0.34631 (17)	0.6994 (2)	0.0323 (6)	
O3	0.2500 (3)	0.43238 (19)	0.8438 (3)	0.0422 (7)	
O4	0.7470 (2)	0.30422 (17)	0.9660 (3)	0.0360 (6)	
C1	0.5707 (3)	0.4021 (2)	0.8231 (3)	0.0248 (7)	
C2	0.6445 (3)	0.4775 (2)	0.8026 (3)	0.0241 (7)	
C3	0.7649 (3)	0.5115 (2)	0.8584 (3)	0.0244 (7)	
H3	0.8189	0.4845	0.9296	0.029*	
C4	0.8018 (3)	0.5868 (2)	0.8045 (3)	0.0281 (7)	
C5	0.7231 (4)	0.6290 (2)	0.7033 (4)	0.0311 (8)	
H5	0.7515	0.6812	0.6713	0.037*	
C6	0.6029 (4)	0.5957 (2)	0.6480 (3)	0.0323 (8)	
H6	0.5475	0.6239	0.5788	0.039*	
C7	0.5685 (3)	0.5196 (2)	0.6990 (3)	0.0275 (8)	
C8	0.4610 (3)	0.4037 (2)	0.7323 (3)	0.0290 (8)	
C9	0.3470 (3)	0.3461 (3)	0.7034 (4)	0.0329 (9)	
H9A	0.3707	0.2879	0.7398	0.040*	
H9B	0.3185	0.3399	0.6099	0.040*	
C10	0.2375 (3)	0.3817 (2)	0.7578 (3)	0.0285 (8)	
C11	0.0149 (4)	0.3741 (3)	0.7474 (4)	0.0439 (10)	
H11A	0.0056	0.4379	0.7412	0.053*	
H11B	0.0246	0.3571	0.8379	0.053*	
C12	−0.1006 (4)	0.3307 (3)	0.6670 (4)	0.0366 (9)	
H12A	−0.1757	0.3436	0.7042	0.044*	
H12B	−0.0868	0.2672	0.6724	0.044*	
C13	−0.1330 (4)	0.3561 (3)	0.5274 (4)	0.0409 (10)	
H13	−0.0564	0.3437	0.4905	0.049*	
C14	−0.2427 (5)	0.2997 (4)	0.4585 (6)	0.080 (2)	
H14A	−0.2175	0.2385	0.4684	0.120*	
H14B	−0.2631	0.3147	0.3677	0.120*	
H14C	−0.3183	0.3093	0.4949	0.120*	
C15	−0.1639 (5)	0.4518 (4)	0.5093 (5)	0.0607 (14)	
H15A	−0.2415	0.4649	0.5408	0.091*	
H15B	−0.1781	0.4664	0.4185	0.091*	
H15C	−0.0921	0.4861	0.5570	0.091*	
C16	0.5774 (4)	0.3837 (3)	1.0723 (4)	0.0367 (9)	
H16A	0.5952	0.3483	1.1499	0.055*	
H16B	0.4876	0.4025	1.0546	0.055*	
H16C	0.6335	0.4347	1.0844	0.055*	

### Atomic displacement parameters (Å^2^)

**Table:**

	*U*^11^	*U*^22^	*U*^33^	*U*^12^	*U*^13^	*U*^23^
I	0.03406 (15)	0.03270 (15)	0.03724 (15)	−0.01078 (10)	0.00567 (10)	0.00262 (11)
S	0.0287 (5)	0.0217 (4)	0.0313 (4)	−0.0019 (3)	0.0046 (4)	−0.0022 (3)
O1	0.0250 (13)	0.0389 (14)	0.0249 (12)	0.0014 (11)	−0.0010 (10)	−0.0019 (11)
O2	0.0220 (12)	0.0414 (15)	0.0322 (13)	0.0005 (11)	0.0032 (10)	−0.0092 (11)
O3	0.0378 (16)	0.0473 (17)	0.0421 (16)	−0.0048 (13)	0.0102 (13)	−0.0208 (14)
O4	0.0285 (14)	0.0327 (14)	0.0448 (16)	0.0031 (11)	0.0030 (12)	0.0004 (12)
C1	0.0253 (17)	0.0234 (17)	0.0256 (17)	0.0004 (14)	0.0052 (14)	−0.0033 (14)
C2	0.0240 (17)	0.0256 (17)	0.0232 (16)	0.0027 (14)	0.0062 (13)	−0.0033 (13)
C3	0.0236 (17)	0.0273 (17)	0.0215 (16)	0.0013 (14)	0.0034 (13)	0.0009 (13)
C4	0.0275 (18)	0.0285 (18)	0.0283 (18)	0.0008 (15)	0.0059 (14)	−0.0023 (15)
C5	0.038 (2)	0.0248 (18)	0.0326 (19)	0.0008 (15)	0.0128 (16)	0.0011 (15)
C6	0.040 (2)	0.0325 (19)	0.0228 (17)	0.0080 (17)	0.0028 (15)	0.0014 (15)
C7	0.0287 (19)	0.0304 (18)	0.0229 (16)	0.0034 (15)	0.0046 (14)	−0.0019 (14)
C8	0.0261 (18)	0.0315 (19)	0.0292 (18)	0.0029 (15)	0.0051 (14)	−0.0072 (15)
C9	0.0237 (18)	0.035 (2)	0.039 (2)	−0.0035 (15)	0.0030 (16)	−0.0122 (16)
C10	0.0270 (18)	0.0294 (18)	0.0273 (17)	0.0008 (15)	0.0019 (14)	0.0000 (15)
C11	0.030 (2)	0.064 (3)	0.039 (2)	0.008 (2)	0.0081 (17)	−0.009 (2)
C12	0.0242 (19)	0.042 (2)	0.045 (2)	0.0004 (17)	0.0108 (17)	−0.0001 (18)
C13	0.035 (2)	0.046 (2)	0.040 (2)	0.0111 (19)	0.0043 (18)	−0.0060 (19)
C14	0.048 (3)	0.091 (5)	0.089 (4)	0.002 (3)	−0.014 (3)	−0.037 (4)
C15	0.059 (3)	0.065 (3)	0.058 (3)	0.025 (3)	0.014 (3)	0.015 (3)
C16	0.049 (2)	0.034 (2)	0.0293 (19)	0.0004 (18)	0.0129 (17)	−0.0031 (16)

### Geometric parameters (Å, °)

**Table:**

I—C4	2.106 (4)	C9—C10	1.515 (5)
I—O4^i^	3.114 (3)	C9—H9A	0.9900
S—O4	1.490 (3)	C9—H9B	0.9900
S—C1	1.762 (4)	C11—C12	1.503 (6)
S—C16	1.786 (4)	C11—H11A	0.9900
O1—C8	1.369 (5)	C11—H11B	0.9900
O1—C7	1.372 (4)	C12—C13	1.516 (6)
O2—C10	1.335 (4)	C12—H12A	0.9900
O2—C11	1.457 (5)	C12—H12B	0.9900
O3—C10	1.195 (4)	C13—C15	1.517 (6)
C1—C8	1.355 (5)	C13—C14	1.518 (7)
C1—C2	1.448 (5)	C13—H13	1.0000
C2—C7	1.390 (5)	C14—H14A	0.9800
C2—C3	1.398 (5)	C14—H14B	0.9800
C3—C4	1.391 (5)	C14—H14C	0.9800
C3—H3	0.9500	C15—H15A	0.9800
C4—C5	1.386 (5)	C15—H15B	0.9800
C5—C6	1.392 (5)	C15—H15C	0.9800
C5—H5	0.9500	C16—H16A	0.9800
C6—C7	1.379 (5)	C16—H16B	0.9800
C6—H6	0.9500	C16—H16C	0.9800
C8—C9	1.484 (5)		
			
C4—I—O4^i^	169.4 (1)	O2—C10—C9	110.6 (3)
O4—S—C1	107.9 (2)	O2—C11—C12	107.3 (3)
O4—S—C16	107.0 (2)	O2—C11—H11A	110.3
C1—S—C16	97.9 (2)	C12—C11—H11A	110.3
C8—O1—C7	106.4 (3)	O2—C11—H11B	110.3
C10—O2—C11	115.1 (3)	C12—C11—H11B	110.3
C8—C1—C2	106.9 (3)	H11A—C11—H11B	108.5
C8—C1—S	123.5 (3)	C11—C12—C13	116.0 (4)
C2—C1—S	129.6 (3)	C11—C12—H12A	108.3
C7—C2—C3	119.6 (3)	C13—C12—H12A	108.3
C7—C2—C1	104.6 (3)	C11—C12—H12B	108.3
C3—C2—C1	135.8 (3)	C13—C12—H12B	108.3
C4—C3—C2	116.8 (3)	H12A—C12—H12B	107.4
C4—C3—H3	121.6	C12—C13—C15	112.1 (4)
C2—C3—H3	121.6	C12—C13—C14	108.8 (4)
C5—C4—C3	122.7 (3)	C15—C13—C14	111.8 (4)
C5—C4—I	119.0 (3)	C12—C13—H13	108.0
C3—C4—I	118.3 (3)	C15—C13—H13	108.0
C4—C5—C6	120.7 (3)	C14—C13—H13	108.0
C4—C5—H5	119.6	C13—C14—H14A	109.5
C6—C5—H5	119.6	C13—C14—H14B	109.5
C7—C6—C5	116.3 (3)	H14A—C14—H14B	109.5
C7—C6—H6	121.8	C13—C14—H14C	109.5
C5—C6—H6	121.8	H14A—C14—H14C	109.5
O1—C7—C6	125.3 (3)	H14B—C14—H14C	109.5
O1—C7—C2	110.9 (3)	C13—C15—H15A	109.5
C6—C7—C2	123.8 (3)	C13—C15—H15B	109.5
C1—C8—O1	111.2 (3)	H15A—C15—H15B	109.5
C1—C8—C9	133.1 (4)	C13—C15—H15C	109.5
O1—C8—C9	115.7 (3)	H15A—C15—H15C	109.5
C8—C9—C10	111.6 (3)	H15B—C15—H15C	109.5
C8—C9—H9A	109.3	S—C16—H16A	109.5
C10—C9—H9A	109.3	S—C16—H16B	109.5
C8—C9—H9B	109.3	H16A—C16—H16B	109.5
C10—C9—H9B	109.3	S—C16—H16C	109.5
H9A—C9—H9B	108.0	H16A—C16—H16C	109.5
O3—C10—O2	124.8 (4)	H16B—C16—H16C	109.5
O3—C10—C9	124.5 (3)		
			
O4—S—C1—C8	144.4 (3)	C3—C2—C7—O1	178.4 (3)
C16—S—C1—C8	−104.9 (3)	C1—C2—C7—O1	−0.3 (4)
O4—S—C1—C2	−36.8 (4)	C3—C2—C7—C6	−1.8 (5)
C16—S—C1—C2	74.0 (4)	C1—C2—C7—C6	179.5 (3)
C8—C1—C2—C7	1.0 (4)	C2—C1—C8—O1	−1.3 (4)
S—C1—C2—C7	−178.0 (3)	S—C1—C8—O1	177.8 (2)
C8—C1—C2—C3	−177.4 (4)	C2—C1—C8—C9	−179.0 (4)
S—C1—C2—C3	3.6 (6)	S—C1—C8—C9	0.0 (6)
C7—C2—C3—C4	−0.4 (5)	C7—O1—C8—C1	1.1 (4)
C1—C2—C3—C4	177.7 (4)	C7—O1—C8—C9	179.3 (3)
C2—C3—C4—C5	2.2 (5)	C1—C8—C9—C10	98.3 (5)
C2—C3—C4—I	−176.4 (2)	O1—C8—C9—C10	−79.4 (4)
O4^i^—I—C4—C5	149.4 (5)	C11—O2—C10—O3	0.4 (5)
O4^i^—I—C4—C3	−31.9 (8)	C11—O2—C10—C9	178.1 (3)
C3—C4—C5—C6	−1.9 (6)	C8—C9—C10—O3	−21.8 (6)
I—C4—C5—C6	176.7 (3)	C8—C9—C10—O2	160.4 (3)
C4—C5—C6—C7	−0.3 (5)	C10—O2—C11—C12	177.6 (3)
C8—O1—C7—C6	179.7 (3)	O2—C11—C12—C13	−64.3 (5)
C8—O1—C7—C2	−0.4 (4)	C11—C12—C13—C15	−61.6 (5)
C5—C6—C7—O1	−178.1 (3)	C11—C12—C13—C14	174.2 (4)
C5—C6—C7—C2	2.1 (5)		

Symmetry codes: (i) −*x*+2, −*y*+1, −*z*+2.

### Hydrogen-bond geometry (Å, °)

**Table:**

*D*—H···*A*	*D*—H	H···*A*	*D*···*A*	*D*—H···*A*
C5—H5···O4^ii^	0.95	2.41	3.310 (5)	159
C16—H16C···O3^iii^	0.98	2.44	3.397 (5)	167

Symmetry codes: (ii) −*x*+3/2, *y*+1/2, −*z*+3/2; (iii) −*x*+1, −*y*+1, −*z*+2.
